# DEVELOPING AN ONLINE TRAINING COURSE FOR OLDER-ADULT VOLUNTEERS TO LEAD DEMENTIA ACTIVITY PROGRAMMING

**DOI:** 10.1093/geroni/igae098.3073

**Published:** 2024-12-31

**Authors:** Lacey DiFranco, Silvia Orsulic-Jeras, Gregg Gorzelle, Sara Powers, Michael Skrajner

**Affiliations:** Benjamin Rose, Cleveland, Ohio, United States; Benjamin Rose, Cleveland, Ohio, United States; Hopeful Aging, Winchester, Massachusetts, United States; Benjamin Rose, Cleveland, Ohio, United States; Hopeful Aging, Winchester, Massachusetts, United States

## Abstract

Research over the last decade has strongly supported the use of music intervention to treat symptoms of dementia. Making Connections Through Music (MCTM), a group music intervention designed for persons living with dementia (PLWD), utilizes older adult volunteers as session leaders, addressing both staff shortages in residential care and the high need for engaging socialization activities. This poster describes the development of MCTM’s online volunteer training course and presents findings from a focus group study (N=7 focus groups) aimed to test course prototypes. Two prototypes of the training were developed and tested with older adult volunteers (n=25; Mage = 76.5; 76% female) and long-term care staff (n=25; Mage = 47.2; 88% female). A thematic analysis yielded a list of recommended changes and identified critical content areas for preparing volunteers to be effective session leaders. Volunteer and staff focus group findings determined a large number of themes including the following: 1) Specific training modules on responsive behaviors, communication challenges, and dementia education; 2) Format preferences such as a self-paced structure and interactive features; 3) Hesitancy with technology-based training; 4) Desire for having supplementary materials available; and 5) Importance of leveraging technology to support long-term program sustainability. Results are currently being utilized in the development of the complete MCTM volunteer online training course. Development of such a training is critical not only for MCTM but also in providing non-professionals the tools to adequately facilitate engaging activities, including group programming, for PLWD across a variety of care settings.

